# Supplementary figures and images for: Combinatorial microRNA Loading into Extracellular Vesicles for Increased Anti-Inflammatory Efficacy
Source: Noncoding RNA. 2022 Oct 21;8(5):71. doi: 10.3390/ncrna8050071 (PMC9611452; doi:10.3390/ncrna8050071)

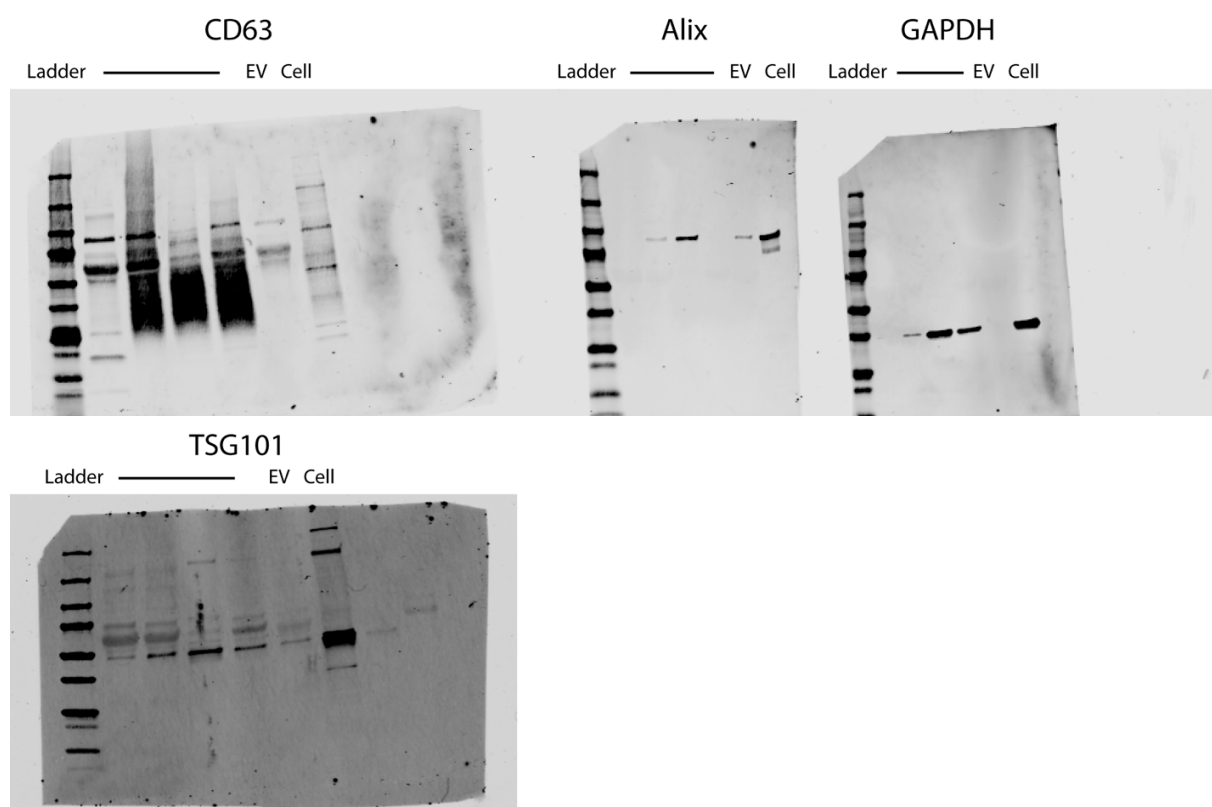

Figure S1: Full immunoblot images related to extracellular vesicle characterization.

Supplement: Supplementary file 1 [file ncrna-08-00071-s001.zip › Figure S1.pdf]
